# Putative *cis*-Regulatory Elements Associated with Heat Shock Genes Activated During Excystation of *Cryptosporidium parvum*

**DOI:** 10.1371/journal.pone.0009512

**Published:** 2010-03-04

**Authors:** Benjamin Cohn, Patricio Manque, Ana M. Lara, Myrna Serrano, Nihar Sheth, Gregory Buck

**Affiliations:** 1 Department of Microbiology and Immunology, Virginia Commonwealth University, Richmond, Virginia, United States of America; 2 Center for the Study of Biological Complexity, Virginia Commonwealth University, Richmond, Virginia, United States of America; Virginia Tech, United States of America

## Abstract

**Background:**

Cryptosporidiosis is a ubiquitous infectious disease, caused by the protozoan parasites *Cryptosporidium hominis* and *C*. *parvum*, leading to acute, persistent and chronic diarrhea worldwide. Although the complications of this disease can be serious, even fatal, in immunocompromised patients of any age, they have also been found to lead to long term effects, including growth inhibition and impaired cognitive development, in infected immunocompetent children. The *Cryptosporidium* life cycle alternates between a dormant stage, the oocyst, and a highly replicative phase that includes both asexual vegetative stages as well as sexual stages, implying fine genetic regulatory mechanisms. The parasite is extremely difficult to study because it cannot be cultured *in vitro* and animal models are equally challenging. The recent publication of the genome sequence of *C. hominis* and *C. parvum* has, however, significantly advanced our understanding of the biology and pathogenesis of this parasite.

**Methodology/Principal Findings:**

Herein, our goal was to identify *cis*-regulatory elements associated with heat shock response in *Cryptosporidium* using a combination of *in silico* and real time RT-PCR strategies. Analysis with Gibbs-Sampling algorithms of upstream non-translated regions of twelve genes annotated as heat shock proteins in the *Cryptosporidium* genome identified a highly conserved over-represented sequence motif in eleven of them. RT-PCR analyses, described herein and also by others, show that these eleven genes bearing the putative element are induced concurrent with excystation of parasite oocysts via heat shock.

**Conclusions/Significance:**

Our analyses suggest that occurrences of a motif identified in the upstream regions of the *Cryptosporidium* heat shock genes represent parts of the transcriptional apparatus and function as stress response elements that activate expression of these genes during excystation, and possibly at other stages in the life cycle of the parasite. Since heat shock and excystation represent a critical step in the development of the infectious sporozoite form of *Cryptosporidium*, these results provide important insight into the pathogenicity of the parasite.

## Introduction

First identified as an opportunistic illness in severely immunosuppressed individuals, cryptosporidiosis is now recognized as a threat to millions of people worldwide. Children under three years old and living in developing countries probably represent the most significant affected population [1], but adults in both developing and developed countries can also be infected [2]. The main etiological agents, *Cryptosporidium hominis* and *C. parvum* [3], cause a severe diarrhea that has been associated with malnutrition, growth retardation, and impaired cognitive development in affected children [4], and death is common in immunocompromised patients [5], [6], [7].

Unlike other apicomplexans, the life cycle of *Cryptosporidium* is completed within a single host, and the parasite is transmitted between hosts via the fecal-oral route. Despite this apparent simplicity, the parasite exhibits a very complex intracellular cycle [8]. The host ingests highly resilient oocysts which excyst in the intestine. The sporozoites released invade intestinal epithelial cells and transform into trophozoites. Trophozoites then progress either to an asexual or sexual meront phase and reinfect additional host epithelial cells or differentiate into male and female gametes, respectively. Gametes unite to form zygotes, which develop into the oocyst and are excreted from the host.

Recently, the genomes of *C. parvum* and *C. hominis* were sequenced, providing the necessary framework to use bioinformatics approaches to explore the biology of this parasite [9], [10]. These sequences have contributed greatly to our understanding of the biology of these important pathogens, often providing insight impossible to obtain using standard laboratory procedures considering the challenges the parasites pose to *in vitro* culture and biochemical or genetic manipulation. For example, although gene regulation in *Cryptosporidium* remains poorly understood, genomics-based bioinformatics strategies provide a means to identify statistically over-represented sequences common to co-regulated genes as putative regulatory motifs, for which corroborating evidence may be provided experimentally [11], [12].

Herein, we combine bioinformatics and molecular approaches to identify putative *cis*-regulatory elements associated with regulation of *C. parvum* heat shock genes. Heat Shock Proteins (HSPs) are highly conserved in all organisms [13]. They are classical molecular chaperones and have been implicated in a broad series of physiological events ranging from the stress response to the immune response in vertebrates [14], [15], [16]. Heat shock has also been found to be an external signal for life cycle events of parasites [17], [18].

*Cryptosporidium* experiences dramatic changes in its environmental conditions, including pH and temperature during its life cycle, and in particular during the infection of the host. These environmental changes seem to elicit a cascade of internal responses in the parasite [15], [19]. We hypothesize that these changes are induced, at least in part, by induction of a stress response including relevant HSPs. Herein, we show that many *C. parvum* genes annotated as HSPs are up-regulated during excystation. Furthermore, we identify putative *cis*-acting regulatory elements in the upstream regions of these genes that may function as heat shock regulatory elements.

## Results and Discussion

Infection by *Cryptosporidium sp.* is initiated when infective oocysts, normally present in the environment, reach the intestine and excyst, releasing invasive sporozoites [20]. Oocysts are exposed to a series of environmental changes including changes in the pH and temperature, which likely serve as signals that trigger the excystation process [18], [21]. Herein, we explored the hypothesis that the *Cryptosporidium* heat shock genes are involved in the excystation process and identify putative *cis*-regulatory elements in their upstream sequences that could be responsible for their co-regulation.

### Heat Shock Genes in *Cryptosporidium*

*Cryptosporidium* heat shock genes were selected by querying our *C. hominis* genome database (www.hominis.mic.vcu.edu) for the presence of genes previously annotated as heat shock genes. We confirmed the putative annotations of these genes as HSPs by selecting only those with highly significant similarity scores to known HSPs from other related organisms using BLASTp (e value<10^−8^) [22]. The gene identifiers for the 12 *C. hominis* genes selected by these criteria are listed in Table 1, as well as their *C. parvum* orthologs, with which subsequent analyses were conducted.

**Table 1:** Heat shock genes of *Cryptosporidium*.

Gene ID *C. parvum*	*C. hominis Ortholog*	Description	Chrom.	Ident.^a^	Upstream^b^	Orientation^c^	Mean Motif Dist.^d^	First Occurrence^e^	Fold Change^f^
cgd2_20	Chro.20010	heat shock 70 (HSP70) protein	2	97%	1755	+	426	99	ND^j^
cgd2_1800	Chro.20195	heat shock 40 kDa protein, putative	2	99%	360	−	427	138	6.19±1.23^g^
cgd2_3230	Chro.20339	heat shock protein DnaJ Pfj2, putative	2	98%	1010	+	593	64	3.93±0.78^g^
cgd2_3330	Chro.20349	APG-1 like HSP70	2	97%	819	−	414	77	6.03±1.80^g^
cgd3_3440	Chro.30389	heat shock protein HSP70	3	98%	661	−	n/a^i^	n/a^i^	ND^j^
cgd3_3770	Chro.30427	Hsp90	3	99%	1098	+	333	72	ND^j^
cgd4_3270	Chro.40370	heat shock 105 kD	4	98%	1175	+	381	166	7.42±1.46^g^
cgd6_1090	Chro.60141	DnaJ(hsp40)	6	98%	629	−	416	406	2.30±0.65^h^
cgd6_2650	Chro.60306	heat shock protein, putative	6	98%	140	−	96	96	4.50±0.83^g^
cgd6_4970	Chro.60573	Hsp60	6	98%	150	−	134	134	3.41±0.79^g^
cgd7_360	Chro.70049	heat shock protein, Hsp70	7	98%	1253	+	585	65	ND^j^
cgd7_3670	Chro.70410	heat shock protein 90	7	97%	217	+	82	60	ND^j^
Average				98%	772		328	125	4.82±1.08

Genes annotated as encoding HSPs in the *C. hominis* and *C. parvum* databases (http://www.hominis.mic.vcu.edu).

a Identity of megablast nucleotide alignment of *C. parvum* and *C. hominis*.

b Upstream Region: number of base pairs between the ATG start codon of the heat shock gene and the closest upstream gene in the *C. hominis* sequence.

c Transcriptional orientation on the chromosome.

d Mean position of motif occurrences from ATG start site of each gene, in base pairs. Values are combined means of hits from AlignACE and MEME algorithms.

e Position of nearest occurrence of motif, as found by either algorithm.

f Fold induction of gene expression, quantified by qRT-PCR. Values shown are ratio of quantity mean values of heat shock to ambient control (37°C/25°C), with standard error.

g P-value<5×10^−6^.

h P-value<0.05.

i Motif not found upstream of cgd3_3440 gene.

j ND; not done.

**Figure 1:**
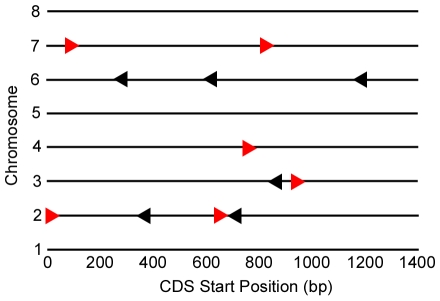
Distribution of *C. parvum* heat shock genes by chromosome. The symbols represent the positions of each of the 12 single copy HSP genes on the eight *C. hominis* chromosomes. Genes oriented (5′ to 32) left to right are indicated by red triangles oriented to the right; genes oriented in the opposite orientation are indicated by black triangles oriented to the left.

Analysis of the physical characteristics of these genes showed that there is no obvious clustering among the eight *Cryptosporidium* chromosomes. Although chromosomes 2 and 6 contain four and three of the 12 heat shock genes analyzed, respectively, the remaining four are spread among chromosomes 3, 4 and 7 (Fig. 1). The orientations of these genes are apparently random (Fig. 1), and the distance from the stop codon of the nearest upstream neighbor, the region likely to contain a transcriptional regulatory apparatus, varied from 140 to 1755 bases (Table 1).

### Putative Heat Shock Gene Regulatory Elements

We considered two possible scenarios when searching for *cis*-regulatory elements: first, that there may exist a known conserved heat shock element motif present in all upstream regions, or alternatively, that a *Cryptosporidium* species-specific motif controls regulation. Initial examination of the upstream regions of each of the 12 putative heat shock genes revealed no classical transcriptional control elements or overrepresented sequences (data not shown). Thus, potential regulatory motifs controlling heat shock genes in *Cryptosporidium* have, to date, remained uncharacterized.

In other organisms, it is well known that transcriptional regulation of heat shock genes is mediated by *cis*-acting heat shock response elements [23]. Thus, we examined the upstream regions of 12 *Cryptosporidium* heat shock genes to identify common sequence motifs that might represent regulatory heat shock response elements. Upstream sequences were extracted, and common sequence motifs–putative *cis*-regulatory motifs–were sought using two alternative implementations of Gibbs-sampling algorithms, AlignACE and MEME [24], [25], as described in the Materials and Methods.

To search for novel heat shock response elements, the upstream regions from the set of putative *C. parvum* heat shock genes (see Table 1) were extracted and analyzed using the Gibbs-sampler, AlignACE. As there is a stochastic element to the algorithm, AlignACE was run iteratively to avoid exclusion of potential high-scoring motifs (not shown). The same set of upstream sequences was analyzed using another Gibbs-sampler, MEME, with the intent of converging upon a biologically relevant motif using multiple algorithms. Consensus sequences were displayed graphically using the WebLogo application [26].

**Figure 2:**
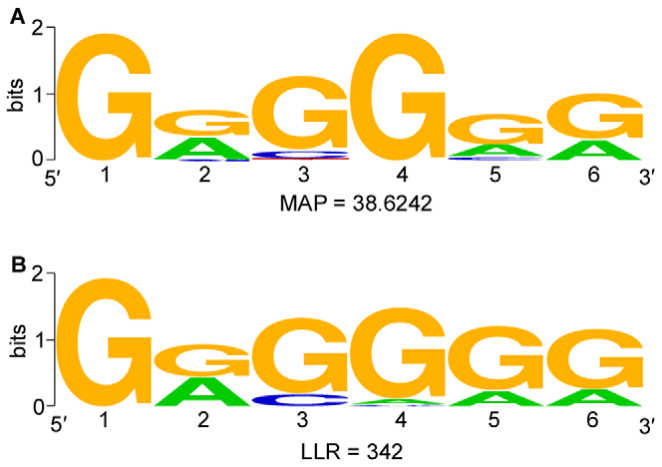
Highest-Scoring motifs found by Gibbs-Sampling algorithms AlignACE and MEME. A and B show the highest-scoring motifs found by AlignACE and MEME, respectively. Maximum *a-priori* Probability (MAP) and Log Likelihood Ratio (LLR) scores are displayed below their respective motifs and are well above cutoffs used in previous studies with the same algorithms. The first occurrence of the motif is located, on average, 125 bp upstream of the transcription start codon (see Table 1).

Our examination of the upstream sequences of the heat shock genes for a conserved motif identified a six base motif, a G-rich sequence represented by the consensus G[G/A][G/C]G[G/A][G/A] (Fig. 2). This sequence appears in the upstream regions of 11 of the 12 putative heat shock genes we considered, and scored well in the respective metrics of each analysis performed (MAP = 38.6242 in AlignACE and LLR = 342 in MEME). On average, it is located 328 bp upstream of the transcription start codon, with the first occurrence at 125 bp, a range typical for heat shock elements in other apicomplexan parasites [27] (Table 1). Only cgd3_3440 did not contain this putative regulatory motif in its upstream region. This gene displays a very conserved HSP70 motif (pfam: <1.0e-180), suggesting that it is a member of the HSP70 gene family–one of the three major HSP gene families [28]. This observation suggests that the HSP70 gene family may, at least in part, fall under a different mechanism of regulation in *Cryptosporidium*, as observed in other systems [28]. Whether a different common transcriptional element regulates all HSP70 genes in *Cryptosporidium* remains unclear, however, as analyzing these genes as a subset with AlignACE yielded no high-scoring (MAP>30) motifs (data not shown).

The putative regulatory motif was also found upstream of orthologous *C. hominis* heat shock genes (Table 1). When upstream regions of these genes were analyzed in the same manner as previously with *C. parvum*, a similar G-rich motif was discovered (data not shown). Though the coding regions between the heat shock genes of these two species are highly conserved (∼98% mean identity), the upstream regions of these genes are slightly more divergent (∼95% mean identity). Regardless, the putative motif was likewise found to be highly conserved between *C. hominis* and *C. parvum*, as aligned motif sites [29] between the two species contained one or fewer sequence mismatches in all cases. Furthermore, if only the occurrence most directly upstream of the gene transcription start codon is considered, the motif is conserved between species in all of the 11 genes in which it is found (data not shown).

To address the question of the prevalence of the motif throughout the genome, the FIMO program [30] was used to search for the motif in three training sets: heat shock upstream regions, coding sequences only and the entire genome. For the heat shock set, presence of the motif was confirmed (as expected) and false discovery rates (FDRs) were very low (q-values between 0.00165 and 0.0334). For the coding sequences, the motif was found by the algorithm, but FDRs were much higher (q-values between 0.171 and 0.500), indicating a greater rate of false positives in these regions. For the entire genome, FDRs were intermediate (q-values between 0.00158 and 0.358), presumably including motifs from both coding and non-coding regions. For nearly any q-value threshold at or below 0.358 (the highest reported by FIMO for the genomic set), more than double the number of occurrences of the motif sequence were found in the genome as a whole than in the coding sequences alone, implying a prevalence of statistically significant motif occurrences in non-coding regions (Fig. S1 and S2).

### Expression of Heat Shock Genes during Excystation

**Figure 3:**
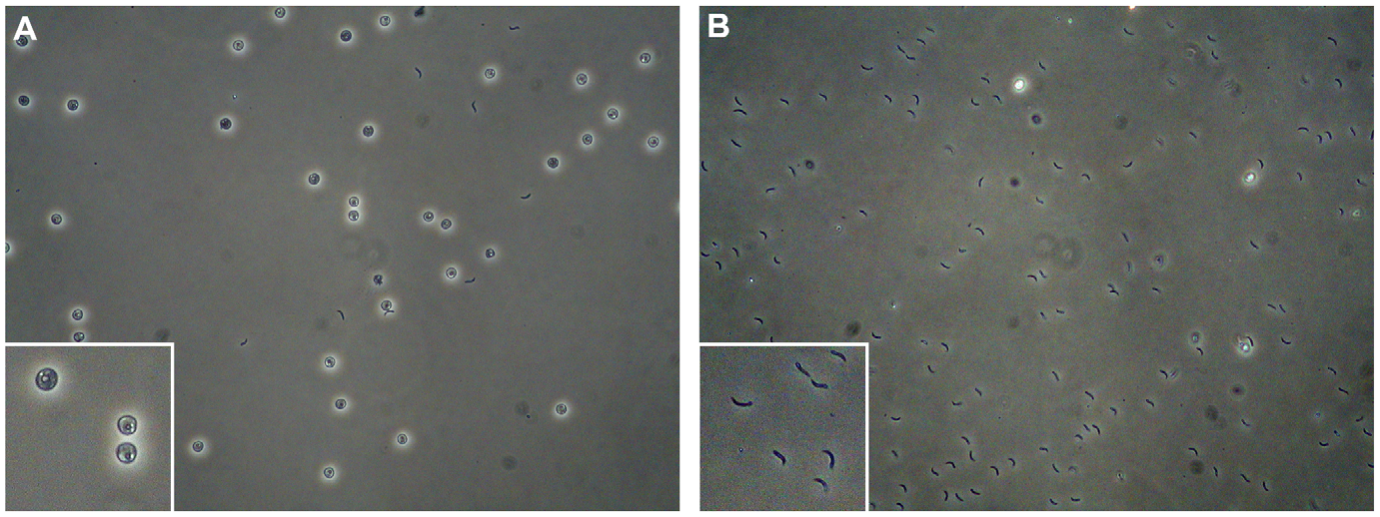
Excystation of *Cryptosporidium parvum* oocysts. To induce exystation, *C. parvum* oocysts were incubated for one hour in excystation medium at: A) 25°C; or B) 37°C (see details in the Materials and Methods), and the resulting parasites were observed by phase microscopy at 40× magnification [inset at ∼100×]. Unexcysted oocysts appear as refractile spheres, sporozoites appear as dark crescent shaped cells, and oocyst ghosts appear as dark black spheres (see insets for close-up).

We applied quantitative real time PCR (qRT-PCR) to measure the relative levels of mRNAs of seven of the eleven motif-bearing heat shock genes before and after excystation (i.e. without or with heat shock, respectively). The remaining four putative heat shock genes have previously been shown to be up-regulated during a similar excystation process in *C. parvum* [19] and therefore were not included in our experiments. Briefly, oocysts were incubated for 60 minutes at 37°C or 25°C in excystation medium and total RNA was extracted as described in the Materials and Methods. As shown in Fig. 3, oocysts incubated in excystation media at 37°C exhibited efficient excystation (∼90%), whereas oocysts incubated in the same medium at 25°C exhibited almost no excystation (<1%). Interestingly, Widmer et al. (2007) showed that the rates of excystation of *C. parvum* incubated in phosphate buffered saline at 0°C or 37°C were very low, indicating that temperature alone does not efficiently induce excystation of *C. parvum* oocysts. Taken together, these observations suggest that the signals required to induce excystation of these parasites are complex and probably involve the synergistic effect of several environmental factors, one of which may be temperature.

The qRT-PCR results, performed in triplicate, showed that each of the seven putative HSP genes tested were significantly (p<0.05) up-regulated during heat-induced excystation (Table 1). These results are consistent with the previous observations [19] showing that the remaining four motif-bearing putative heat shock proteins are also up-regulated in excysted sporozoites. Gene expression analysis using comprehensive *Cryptosporidium* microarrays shows that, under identical conditions, while many genes are up-regulated besides those mentioned herein, others are unaffected or even down-regulated (Serrano et al., unpublished). Thus, we conclude that the physiological conditions that induce excystation also clearly trigger molecular cascades that lead to the coordinated transcription of these heat shock proteins, though this likely represents only part of a more complicated transcriptional program during this phase of the parasite's life cycle.

Currently, mechanisms of gene regulation in *Cryptosporidium* remain poorly understood. Traditional biochemical and genetic approaches to dissecting these mechanisms are largely unavailable due to the difficulty of manipulation of the parasite both *in vitro* and *in vivo*; e.g., the parasite cannot be continuously cultivated and no genetic system is available for it. The genome sequences of *C. hominis* and *C. parvum* revealed relatively few classical transcription factors (see www.hominis.mic.vcu.edu). Moreover, although ubiquitous transcriptional signals such as TATA boxes were found, the relatively high AT content of intergenic sequences (∼70%) and the absence of data regarding transcriptional starting points make it challenging to define their role in the regulation of transcription. Recently, however, putative *cis*-acting regulatory elements associated with the genes in other metabolic pathways have been identified using *in silico* approaches [11], [12]. Thus, informatics-based strategies are likely to provide important insights into the mechanisms of gene expression in this challenging model.

Here, using a combination of molecular and *in silico* strategies, we have studied expression of a panel of putative heat shock genes of *Cryptosporidium*. Our data demonstrate a concerted up-regulation of seven putative heat shock genes during the process of excystation, which in this case involves a temperature shock and other environmental changes. Since previous data indicate a similar induction of four additional putative heat shock genes [19], at least 11 of the 12 putative heat shock genes are up-regulated during excystation. *In silico* analysis of the upstream regions of these putative heat shock genes using motif-finding algorithms identified a well-conserved sequence motif, for which we now propose a putative role in heat shock regulation. The biological role of the putative heat shock or stress regulatory element remains to be verified *in vivo*.

## Materials and Methods

### Parasite Source

Iowa strain *C. parvum* oocysts used in this study were purchased from the Sterling Parasitology Laboratory in Tuscon, AZ. Oocysts were purified using discontinuous sucrose and cesium chloride centrifugation gradients and shipped in an antibiotic solution containing 0.01% Tween 20, 100 U penicillin and 100 µg of gentamicin per ml. Purified oocysts were stored at 4°C for less than 30 days prior to use.

### Heat Shock Treatment

1×10^8^ *C. parvum* oocysts were incubated on ice in a solution containing 40% bleach in PBS. Oocysts were then washed three times (5,000×*g* for 4 minutes at 4°C) with Hanks' Balanced Salt Solution, transferred to “excystation medium” (0.75% Sodium Taurocholate and 0.25% Trypsin in PBS or Hanks' medium [31]), and incubated at either 25°C (no heat shock) or 37°C (heat shock) for one hour prior to RNA extraction.

### RNA Extraction

RNA from parasites incubated at 25°C (>99% oocysts) was extracted using TRIZOL (Invitrigen), with the addition of 100 µg of glycogen (Boehringer Mannhein) and 100 U of RNase inhibitor (SUPERase-In from Ambion). RNA from parasites incubated at 37°C (>90% sporozoites) was extracted using the RNAqueous system (Ambion) with the addition of 100 µg of glycogen and 100 U of RNase inhibitor, as described by the manufacturer.

### Real-Time Quantitative RT-PCR

RNAs were treated with TURBO DNA-free DNAse (Ambion) following manufacturers instructions and used for Real-Time RT-PCR analysis using TaqMan™ (ABI) technology. Primers and probes specific for putative heat shock genes (Table S1) were designed using Primer Express® version 2.0 (ABI). For each target, forward and reverse primers and an internal probe were synthesized. Probes were synthesized with 5′ end linked FAM (6-carboxyfluoresceine) and 3′ end fluorescent TAMRA (6-carboxytetramethylrhodamine) dyes. Amplification and analysis was performed in an ABI 7900HT instrument essentially as described by the manufacturer, and a parallel quantification of the small subunit rRNA transcripts was performed as a normalization control. A “no reverse transcriptase” control experiment was performed to detect DNA contamination.

Quantity mean (QM), a relative measure of gene expression, was determined as a function of PCR cycle count (Ct), as extrapolated from a standard curve of known concentrations (i.e., quantities of input RNA) for each HSP gene. To determine the comparative gene expression level, we first calculated the statistical significance (p-value) between the average QM of each gene over all experiments at 37°C versus 25°C, using Student's T-test (Table 1). Genes were then normalized by dividing the QM of each HSP gene by that of the *C. parvum* small subunit ribosomal (18S) gene, and fold change determined by calculating the ratio between normalized gene expressions of each gene at 37°C and 25°C. Standard error was calculated by propagating the standard deviation of each QM throughout subsequent calculations.

### Identification of Conserved Motifs in the Upstream Regions of Heat Shock Genes

Upstream regions (see Table 1) extracted from the set of putative *Cryptosporidium* heat shock genes were analyzed using the Gibbs-sampler, AlignACE [^24^], where parameters were set to 10 aligned columns, 10 expected sites and GC% = 0.27 (the fractional GC content for these upstream regions in *C. parvum*). Though most motifs contain fewer than ten bases, we chose more inclusive parameters (“10 aligned columns,” above) so as not to inadvertently cut information-rich bases out of a putative motif. Indeed, varying this parameter did not yield significantly different motifs and it is the 6 bp information-rich segment of the longer motif that we report in this study (Fig. 2A). The parameter “number of expected sites” refers to the number of expected occurrences of the motif that may be found among the training set (not, for example, to the number of genes in which it may be found), and is not highly stringent. For instance, AlignACE allows for more than 10 occurrences, should they exist in the input sequence. Given the stochastic element to the algorithm, AlignACE was run 10 times with the same input sequences, and the top motif from all runs selected. The same set of upstream sequences was also analyzed using MEME [^25^] in TCM mode, and trained on the intergenic region of these genes and with minimum and maximum widths set at 6 and 50 bases, respectively. The results of both programs were represented graphically using the WebLogo application [26] (Fig. 2).

To examine conservation of the putative motif between *C. parvum* and *C. hominis*, the same analysis was performed on the orthologous set of putative heat shock genes in *C. hominis*. The motif occurrences of either species were then mapped onto a BLAST [29] alignment of the two upstream regions to see where they coincided, if at all.

To address prevalence of the motif within the *C. parvum* genome, the FIMO program [30] was used to search for a position-specific scoring matrix (PSSM) representing the motif within three sets: heat shock upstream regions (positive control), coding regions only, and the entire genome.

## Supporting Information

Figure S1 **Occurrences of motif identified by FIMO.** FIMO identified occurrences of the motif in heat shock upstream regions (green triangles), genomic contigs (blue diamonds) and coding regions only (red squares). Abscissa indicates number of motif occurrences found.
(2.04 MB TIF)

Figure S2 **Segregation of FIMO-identified motifs by false discovery rate.** Enlarged lower left-hand portion of Figure S2 shows that false discovery rates for the motif, indicated here by q-value, are highest for coding regions (red squares), followed by genomic contigs (blue diamonds) and the positive control, heat shock upstream regions (green triangles).
(2.01 MB TIF)

Table S1 Probes and Primers used in quantitative real-time RT-PCR experiments. Gene-specific primers and probes used in RT PCR experiments to show the up regulation of the indicated heat shock genes.
(0.04 MB DOC)
