# Supplementary figures and images for: Putative cis-Regulatory Elements Associated with Heat Shock Genes Activated During Excystation of Cryptosporidium parvum
Source: PLoS One. 2010 Mar 4;5(3):e9512. doi: 10.1371/journal.pone.0009512 (PMC2832001; doi:10.1371/journal.pone.0009512)

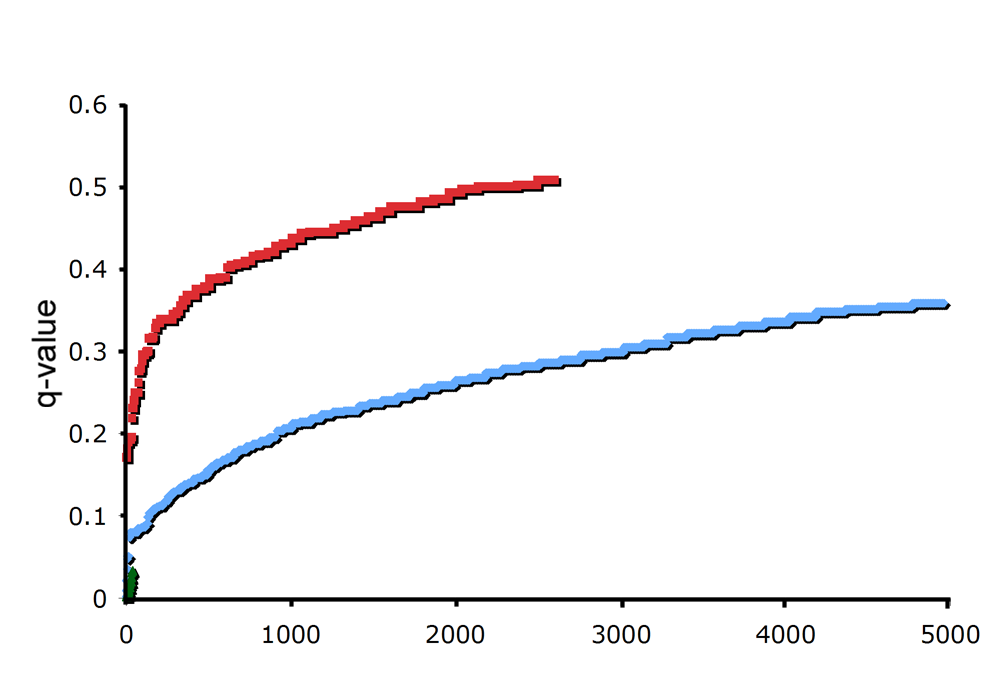

Supplement: Figure S1 — Occurrences of motif identified by FIMO. FIMO identified occurrences of the motif in heat shock upstream regions (green triangles), genomic contigs (blue diamonds) and coding regions only (red squares). Abscissa indicates number of motif occurrences found. (2.04 MB TIF) [file pone.0009512.s001.tif]

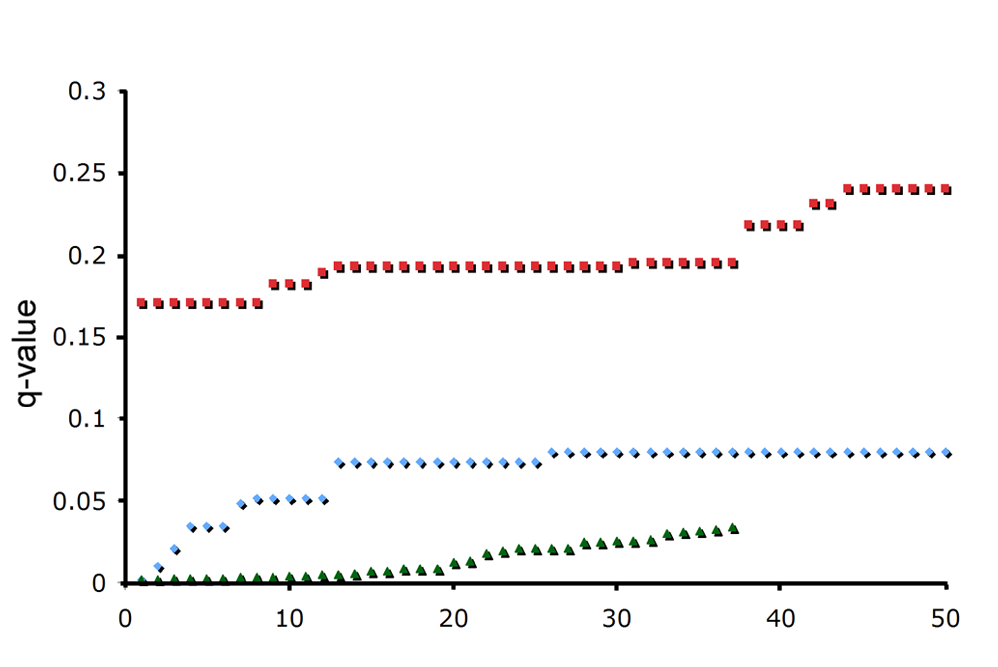

Supplement: Figure S2 — Segregation of FIMO-identified motifs by false discovery rate. Enlarged lower left-hand portion of Figure S2 shows that false discovery rates for the motif, indicated here by q-value, are highest for coding regions (red squares), followed by genomic contigs (blue diamonds) and the positive control, heat shock upstream regions (green triangles). (2.01 MB TIF) [file pone.0009512.s002.tif]
