# Supplementary material for: Putative cis-Regulatory Elements Associated with Heat Shock Genes Activated During Excystation of Cryptosporidium parvum
Source: PLoS One. 2010 Mar 4;5(3):e9512. doi: 10.1371/journal.pone.0009512 (PMC2832001; doi:10.1371/journal.pone.0009512)
Supplement: Table S1 — Probes and Primers used in quantitative real-time RT-PCR experiments. Gene-specific primers and probes used in RT PCR experiments to show the up regulation of the indicated heat shock genes. (0.04 MB DOC) [file pone.0009512.s003.doc]

##### Supporting Tables

**Table S1. Probes and Primers used in quantitative real-time RT-PCR experiments.**

| **Gene** |  | **Sequence** | **Start^a^ (bp)** | **Stop^b^ (bp)** | **Tm^c^** |
| --- | --- | --- | --- | --- | --- |
| cgd2_1800 | Forward Primer | 5’ GAATGTTCAGACAAGTACACATGTCATC 3’ | 383 | 410 | 59 |
|  | Reverse Primer | 5’ ATGGGACTTCGTGCGATCTT 3’ | 466 | 447 | 58 |
|  | Probe | 5’ ACGCATAATGGGCGTTCTTCAACTAGACAC 3’ | 412 | 441 | 68 |
| cgd6_1090 | Forward Primer | 5’ TGTCAGTGGAGCAAGAACAACTAAA 3’ | 294 | 318 | 59 |
|  | Reverse Primer | 5’ CGGATTAGTTCCTTGTCTTGCA 3’ | 375 | 354 | 58 |
|  | Probe | 5’ CCCACCTCCAAATAATGACCCATCGA 3’ | 320 | 345 | 68 |
| cgd6_4970 | Forward Primer | 5’ GAATGTGGCAATATCAACAAACG 3’ | 321 | 343 | 58 |
|  | Reverse Primer | 5’ ATCCTGCATCAACTTTCTCACAAC 3’ | 427 | 404 | 58 |
|  | Probe | 5’ CACAACTACAGCCACCGTGTTAGCAAGAGC 3’ | 360 | 389 | 70 |
| cgd2_3330 | Forward Primer | 5’ CCAGGAAGAGGAATTGAGATAGTTTT 3’ | 139 | 164 | 58 |
|  | Reverse Primer | 5’ TATAGAAGGAGATGAAGTGCTGAATGA 3’ | 228 | 202 | 59 |
|  | Probe | 5’ CTCATAGCCAACGAAAGACAGCGACAGC 3’ | 170 | 197 | 69 |
| cgd4_3270 | Forward Primer | 5’ TGGTAGAGACGGAGAAACTTTATTCAT 3’ | 233 | 259 | 59 |
|  | Reverse Primer | 5’ TTGATTGCCCTTATAGTTTACTCTGAAC 3’ | 324 | 297 | 58 |
|  | Probe | 5’ CGGAAATCACAACATGCGAAAACGGTAAC 3’ | 263 | 291 | 70 |
| cgd2_3230 | Forward Primer | 5’ CCCCACATGGAAGCTTCAA 3’ | 1066 | 1084 | 58 |
|  | Reverse Primer | 5’ TCTGTGGCTTTTCCTCCAACTT 3’ | 1163 | 1142 | 59 |
|  | Probe | 5’ ACCCGCAAATCCCCCTTATATTCTCTCAGT 3’ | 1086 | 1115 | 68 |
| cgd6_2650 | Forward Primer | 5’ GGCTCAGGTTTTGGAAATATGC 3’ | 110 | 131 | 59 |
|  | Reverse Primer | 5’ GAGACCCCTCCTTGGAATCC 3’ | 186 | 167 | 58 |
|  | Probe | 5’ AGCTTTTCATCTTCCTCATTCAGCTCTGGG 3’ | 134 | 163 | 69 |

^a^ Start position of the primer or internal probe, with respect to the coding region of the gene.

^b^ Similarly, the stop position of the primer or probe.

^c^ Melting temperature of oligonucleotide.
